# Supplementary material for: Development and application of a high-content virion display human GPCR array
Source: Nat Commun. 2019 Apr 30;10:1997. doi: 10.1038/s41467-019-09938-9 (PMC6491619; doi:10.1038/s41467-019-09938-9)
Supplement: Supplementary file 1 — Supplementary Information [file 41467_2019_9938_MOESM1_ESM.pdf]

Development and Application of a High-Content Virion Display Human GPCR Array

Syu et al.

Supplementary files

SRIF-14 binding	k_a ($M^{-1}s^{-1}$)	k_d (s^{-1})	K_D (μM)
SSTR2 virion	$(4.0 \pm 0.0) \times 10^{3}$	$(4.5 \pm 0.1) \times 10^{-5}$	$(11.2 \pm 0.1) \times 10^{-3}$
GABBR2 virion	$(9.6 \pm 0.3) \times 10^{2}$	$(3.7 \pm 0.2) \times 10^{-4}$	0.39 ± 0.01
NTSR1 virion	$(2.2 \pm 0.1) \times 10^{3}$	$(5.7 \pm 0.1) \times 10^{-3}$	2.6 ± 0.1
KISS1R virion	362.0 ± 1.0	$(9.1 \pm 0.1) \times 10^{-3}$	25.0 ± 0.1
K082 virion	No binding	No binding	No binding

Supplementary Table 1. The binding kinetics of SIRF-14 to SSTR2, NTSR1, KISS1R, GABBR2, and K082 virions.

Supplementary Table 2. List of cloned VirD-GPCR.

Symbol	Uniprot	DNA length
HTR1A	P08908	1269
HTR1B	P28222	1170
HTR1D	P28221	1134
HTR1E	P28566	1098
HTR1F	P30939	1101
HTR2B	P41595	1446
HTR2C	P28335	1374
HTR4	Q13639	1167
HTR5A	P47898	1074
HTR6	P50406	1320
HTR7	P34969	1299
CHRM1	P11229	1383
CHRM2	P08172	1401
CHRM3	P20309	1773
CHRM4	P08173	1440
CHRM5	P08912	1596
ADORA1	P30542	978
ADORA2A	P29274	1239
ADORA2B	P29275	996
ADORA3	P33765	954
BAI1	O14514	4752
BAI3	O60242	4566
CD97	P48960	2226
ELTD1	Q9HBW9	1821
EMR1	Q14246	2463
EMR2	Q9UHX3	2472
EMR3	Q9BY15	1926
EMR4P	Q86SQ3	696
GPR56	Q9Y653	2082
GPR64	Q8IZP9	2850
GPR97	Q86Y34	1650
GPR110	Q5T601	657
GPR111	Q8IZF7	2124

Symbol	Uniprot	DNA length
GPR50	Q13585	1854
GPR52	Q9Y2T5	1083
GPR55	Q9Y2T6	960
GPR61	Q9BZJ8	1356
GPR62	Q9BZJ7	1107
GPR63	Q9BZJ6	1257
GPR65	Q8IYL9	1014
GPR68	Q15743	1095
GPR75	O95800	1623
GPR78	Q96P69	1092
GPR82	Q96P67	1011
GPR83	Q9NYM4	1269
GPR84	Q9NQS5	1191
GPR85	P60893	1113
GPR87	Q9BY21	1074
GPR88	Q9GZN0	1152
GPR101	Q96P66	1527
GPR119	Q8TDV5	1008
GPR132	Q9UNW8	1143
GPR135	Q8IZ08	1449
GPR139	Q6DWJ6	1059
GPR141	Q7Z602	918
GPR142	Q7Z601	1386
GPR146	Q96CH1	999
GPR148	Q8TDV2	1041
GPR149	Q86SP6	2193
GPR150	Q8NGU9	1302
GPR151	Q8TDV0	1260
GPR152	Q8TDT2	1410
GPR153	Q6NV75	1830
GPR160	Q9UJ42	1017
GPR161	Q8N6U8	1590
GPR162	Q16538	912

Symbol	Uniprot	DNA length
HCAR3	P49019	1164
KISS1R	Q969F8	1195
LTB4R	Q15722	1056
LTB4R2	Q9NPC1	1167
CYSLTR1	Q9Y271	1014
CYSLTR2	Q9NS75	1041
OXER1	Q8TDS5	1272
LPAR1	Q92633	1095
LPAR2	Q9HBW0	1056
LPAR3	Q9UBY5	1062
LPAR4	Q99677	1110
LPAR5	Q9H1C0	1119
LPAR6	P43657	1035
S1PR1	P21453	1149
S1PR2	O95136	1059
S1PR3	Q99500	1134
S1PR4	O95977	1152
S1PR5	Q9H228	1197
MCHR1	Q99705	1266
MCHR2	Q969V1	1023
MC1R	Q01726	954
MC2R	Q01718	894
MC3R	P41968	1080
MC4R	P32245	999
MC5R	P33032	978
MTNR1A	P48039	1050
GRM1	Q13255	3582
GRM2	Q14416	2619
GRM3	Q14832	2640
GRM4	Q14833	2736
GRM7	Q14831	2748
GRM8	O00222	2727
MLNR	O43193	1236

GPR112	Q8IZF6	9240
GPR113	Q8IZF5	3237
GPR114	Q8IZF4	1587
GPR115	Q8IZF3	2085
GPR116	Q8IZF2	3612
GPR123	Q86SQ6	3837
GPR125	Q8IWK6	3963
GPR126	Q86SQ4	3669
GPR128	Q96K78	2394
LPHN1	O94910	4407
ADRA1A	P35348	1401
ADRA1B	P35368	1563
ADRA1D	P25100	1716
ADRA2A	P08913	1353
ADRA2B	P18089	1350
ADRA2C	P18825	1389
ADRB2	P07550	1242
ADRB3	P13945	1227
AGTR1	P30556	1080
AGTR2	P50052	1092
APLNR	P35414	1140
GPBAR1	Q8TDU6	990
GRPR	P30550	1155
BRS3	P32247	1197
BDKRB1	P46663	1062
BDKRB2	P30411	1176
CALCR	P30988	1425
CALCRL	Q16602	1386
CASR	P41180	3237
CNR1	P21554	1419
CNR2	P34972	1083
CMKLR1	Q99788	1116
CCR1	P32246	1068
CCR2	P41597	1122
CCR3	P51677	1068
CCR4	P51679	1083
CCR5	P51681	1059

GPR171	O14626	960
GPR173	Q9NS66	1119
GPR176	Q14439	1545
GPR182	O15218	1215
GPR183	P32249	1083
LGR4	Q9BXB1	2781
LGR5	O75473	2721
LGR6	Q9HBX8	2487
MAS1	P04201	978
MAS1L	P35410	1134
MRGPRD	Q8TDS7	966
MRGPRE	Q86SM8	933
MRGPRF	Q96AM1	1029
MRGPRX2	Q96LB1	993
MRGPRX3	Q96LB0	966
MRGPRX4	Q96LA9	966
OPN3	Q9H1Y3	1206
OPN4	Q9UHM6	1434
OPN5	Q6U736	1062
P2RY8	Q86VZ1	1080
P2RY10	O00398	1020
TAAR2	Q9P1P5	921
TAAR5	O14804	1014
TAAR6	Q96RI8	1038
TAAR8	Q969N4	1029
TAAR9	Q96RI9	1044
GPR156	Q8NFN8	2442
GPR158	Q5T848	3645
GPRC5A	Q8NFJ5	1074
GPRC5B	Q9NZH0	1212
GPRC5C	Q9NQ84	1362
GPRC5D	Q9NZD1	1038
FZD3	Q9NPG1	1998
FZD4	Q9ULV1	1614
FZD5	Q13467	1758
FZD6	O60353	2121
FZD7	O75084	1722

NMUR1	Q9HB89	1278
NMUR2	Q9GZQ4	1248
NPFFR1	Q9GZQ6	1290
NPFFR2	Q9Y5X5	1263
NPSR1	Q6W5P4	1131
NPBWR1	P48145	984
NPBWR2	P48146	999
NPY1R	P25929	1155
NPY2R	P49146	1143
NPY4R	P50391	1128
NPY5R	Q15761	1338
NPY6R	Q99463	873
NTSR1	P30989	1254
NTSR2	O95665	1233
OPRD1	P41143	1116
OPRK1	P41145	1140
OPRM1	P35372	1203
OPRL1	P41146	1113
HCRTR1	O43613	1278
HCRTR2	O43614	1335
GPR107	Q5VW38	1656
GPR137	Q96N19	1191
OR51E1	Q8TCB6	954
TPRA1	Q86W33	717
GPR143	P51810	1275
GPR157	Q5UAW9	1005
OXGR1	Q96P68	1011
P2RY1	P47900	1119
P2RY2	P41231	1131
P2RY4	P51582	1095
P2RY6	Q15077	984
P2RY11	Q96G91	1116
P2RY12	Q9H244	1029
P2RY13	Q9BPV8	1002
P2RY14	Q15391	1014
PTH1R	Q03431	1779
PTH2R	P49190	1650

CCR6	P51684	1125
CCR7	P32248	1137
CCR8	P51685	1068
CCR9	P51686	1074
CCR10	P46092	1089
CXCR1	P25024	1053
CXCR2	P25025	1083
CXCR3	P49682	1104
CXCR4	P61073	1059
CXCR5	P32302	1119
CXCR6	O00574	1029
CX3CR1	P49238	1068
XCR1	P46094	1002
ACKR1	Q16570	1011
ACKR2	O00590	1155
ACKR3	P25106	1089
ACKR4	Q9NPB9	1053
CCRL2	O00421	1035
CCKBR	P32239	1344
GPR1	P46091	1068
GPR3	P46089	993
GPR4	P46093	1089
GPR6	P46095	1086
GPR12	P47775	1005
GPR15	P49685	1083
GPR17	Q13304	1020
GPR18	Q14330	993
GPR19	Q15760	1245
GPR20	Q99678	1074
GPR21	Q99679	1050
GPR22	Q99680	1299
GPR25	O00155	1083
GPR26	Q8NDV2	1011
GPR27	Q9NS67	1125
GPR31	O00270	960
GPR32	O75388	1071
GPR34	Q9UPC5	1146

FZD8	Q9H461	2082
FZD9	O00144	1773
FZD10	Q9ULW2	1746
SMO	Q99835	2364
C3AR1	Q16581	1449
C5AR1	P21730	1053
C5AR2	Q9P296	1014
CRHR1	P34998	1248
CRHR2	Q13324	1236
DRD1	P21728	1341
DRD2	P14416	1329
DRD3	P35462	1200
DRD4	P21917	1257
DRD5	P21918	1434
EDNRA	P25101	1284
EDNRB	P24530	1329
GPER1	Q99527	1128
FPR1	P21462	1053
FPR2	P25090	1056
FPR3	P25089	1062
FFAR1	O14842	900
FFAR2	O15552	990
FFAR3	O14843	1038
GABBR1	Q9UBS5	2532
GABBR2	O75899	2652
GALR1	P47211	1080
GALR2	O43603	1161
GALR3	O60755	1104
GHSR	Q92847	870
GHRHR	Q02643	1269
GIPR	P48546	1398
GLP1R	P43220	1389
GLP2R	O95838	1662
GCGR	P47871	1434
SCTR	P47872	1323
FSHR	P23945	2085
LHCGR	P22888	2097

QRFPR	Q96P65	1068
PTAFR	P25105	1026
PROKR1	Q8TCW9	1179
PROKR2	Q8NFJ6	1152
PRLHR	P49683	1113
PTGDR	Q13258	1080
PTGDR2	Q9Y5Y4	1185
PTGER1	P34995	1209
PTGER2	P43116	1077
PTGER3	P43115	1125
PTGER4	P35408	1467
PTGFR	P43088	1080
PTGIR	P43119	1161
TBXA2R	P21731	1029
F2R	P25116	1278
F2RL1	P55085	1194
F2RL2	O00254	1125
F2RL3	Q96RI0	1155
RXFP1	Q9HBX9	2271
RXFP2	Q8WXD0	2262
RXFP3	Q9NSD7	1410
RXFP4	Q8TDU9	1122
SSTR1	P30872	1176
SSTR2	P30874	1110
SSTR3	P32745	1254
SSTR4	P31391	1164
SSTR5	P35346	1092
SUCNR1	Q9BXA5	1005
TACR1	P25103	1221
TACR2	P21452	1194
TACR3	P29371	1395
TRHR	P34981	1194
TAAR1	Q96RJ0	1020
UTS2R	Q9UKP6	1167
AVPR1A	P37288	1257
AVPR1B	P47901	1272
AVPR2	P30518	1113

GPR35	Q9HC97	927
GPR37	O15354	1839
GPR37L1	O60883	1446
GPR39	O43194	1362
GPR42	O15529	1038
GPR45	Q9Y5Y3	1116

TSHR	P16473	2292
GNRHR	P30968	984
HRH1	P35367	1464
HRH2	P25021	1194
HRH4	Q9H3N8	1170
HCAR1	Q9BXC0	1041

OXTR	P30559	1167
ADCYAP1R1	P41586	1488
VIPR1	P32241	1371
VIPR2	P41587	1314

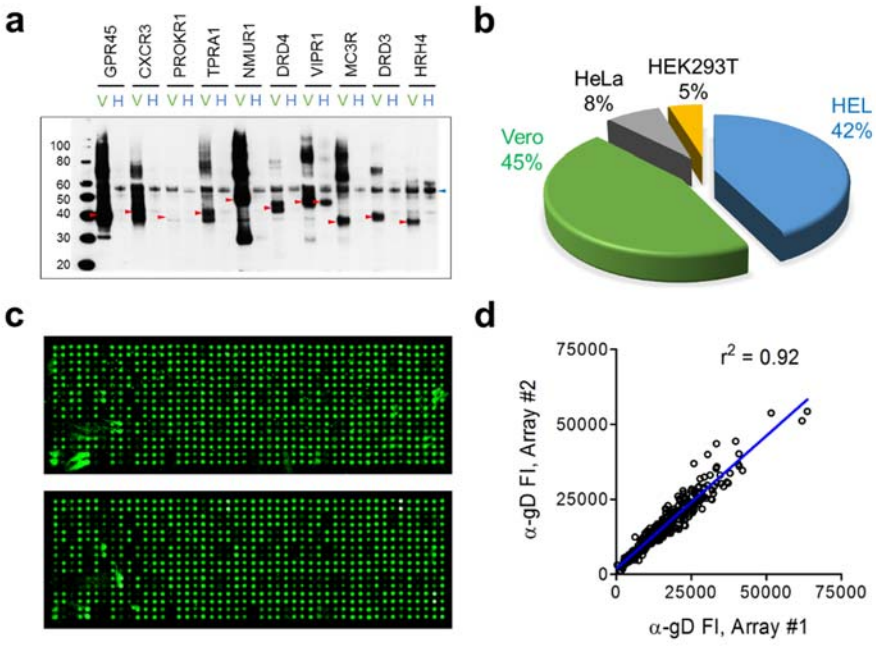

Supplementary Fig. 1 Cell line selection for virion production and the reproducibility of the GPCR-VirD arrays. **a** Anti-V5 IB analysis of VirD-GPCRs expressed in HEL or Vero cells. **b** Distribution of cell lines used to achieve optimal expression of VirD-GPCRs. **c, d** The reproducibility of two VirD-GPCR arrays is illustrated in array images (**c**) and the scatter plot (**d**) of anti-gD staining. Pearson's and Spearman's correlation coefficients were provided.

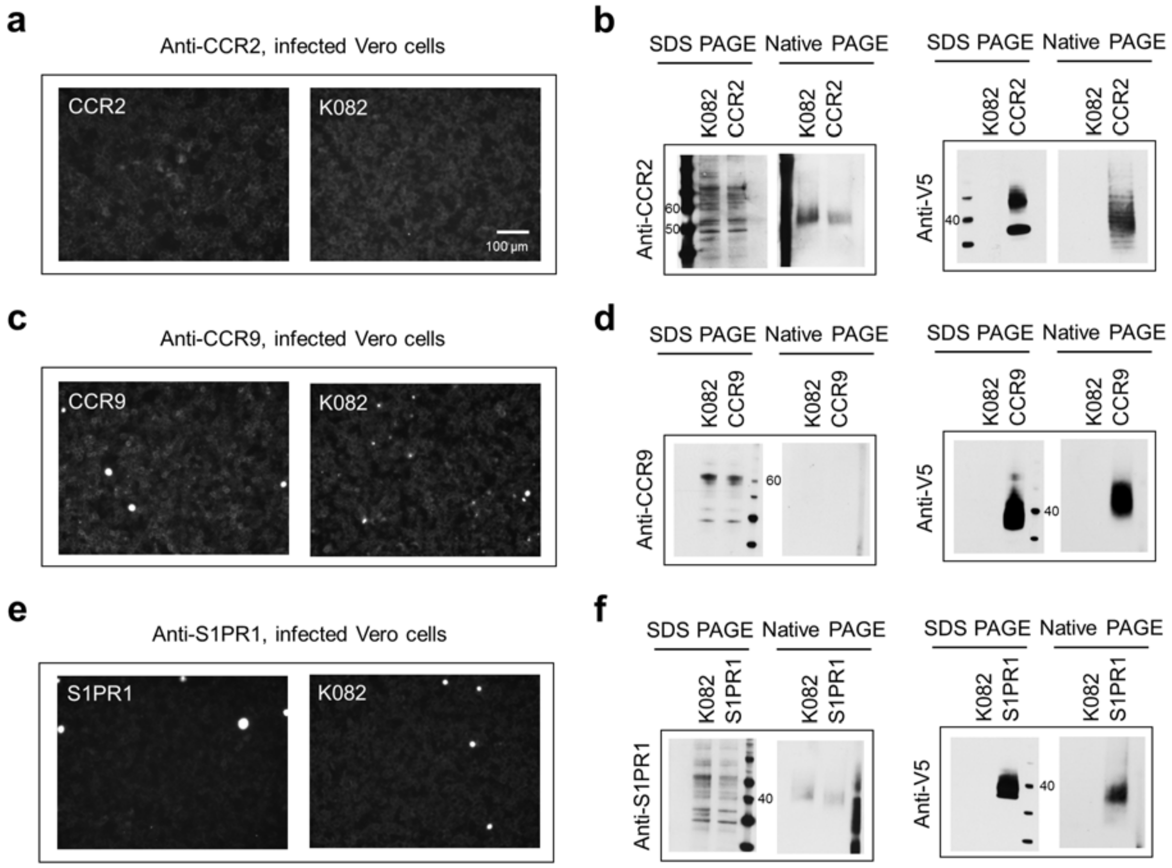

Supplementary Fig. 2 IFA and IB analyses with the commercial mAbs that failed to recognize their intended targets on the VirD-GPCR arrays. **a** IFA staining of anti-CCR2 in infected Vero cells. K082-infected cells are shown as a negative control. **b** IB analysis of anti-CCR2 in the cell lysate of infected Vero cells under the native or denatured condition. **c, d** Anti-CCR9 failed to recognize VirD-DRD1-infected cells. **e, f** Anti-S1PR1 failed to recognize VirD-DRD1-infected cells.

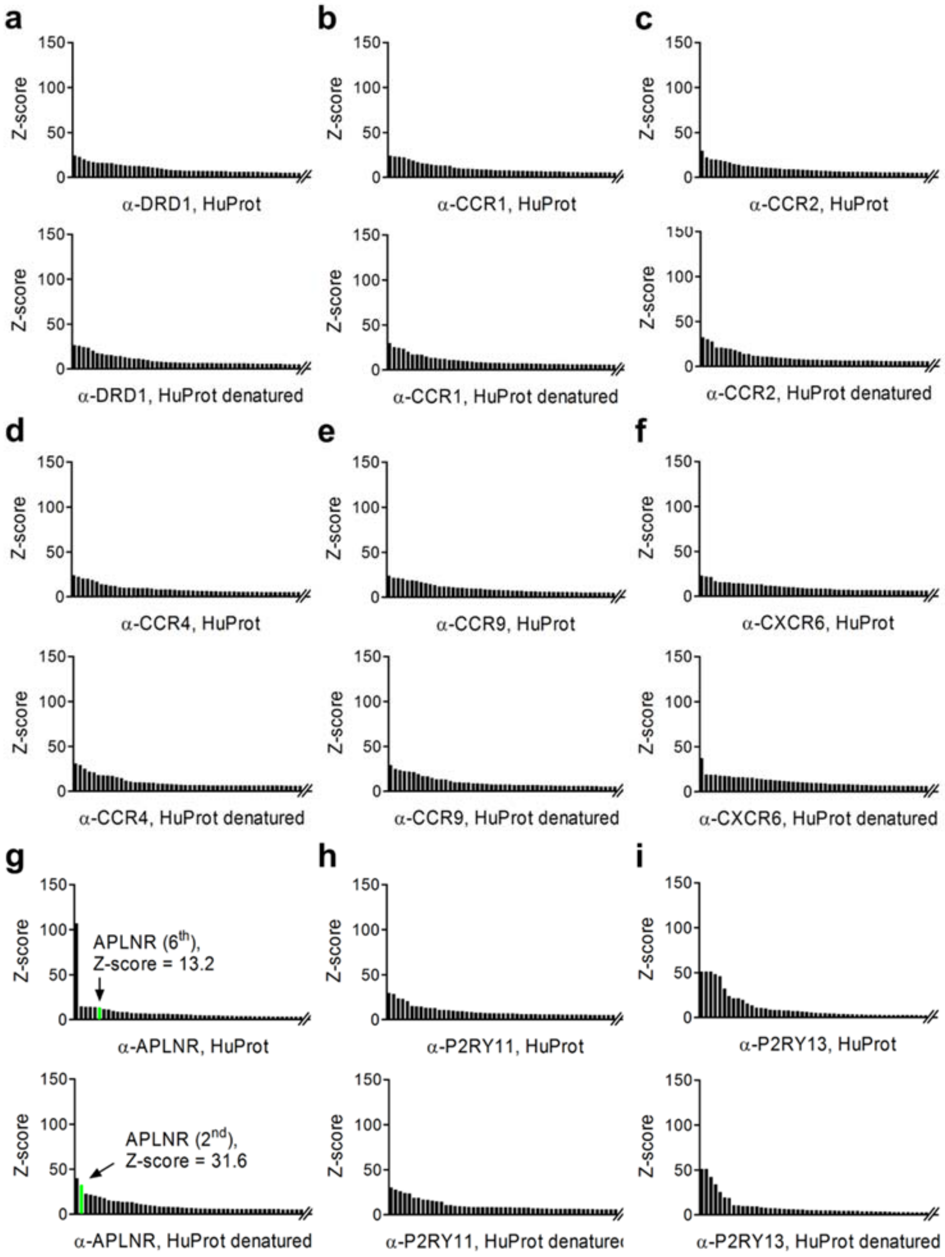

Supplementary Fig. 3 Histogram analysis of binding signals obtained on HuProt arrays with the nine commercial mAbs that failed to recognize their intended targets on the VirD-GPCR arrays. **a-i** HuProt arrays were assessed in either native or denatured form (9 M urea treatment). Top 50 Z-scores of each binding assay were showed in the histograms. None of the tested mAbs recognized their intended targets as the top 1 targets under either native or denatured conditions.

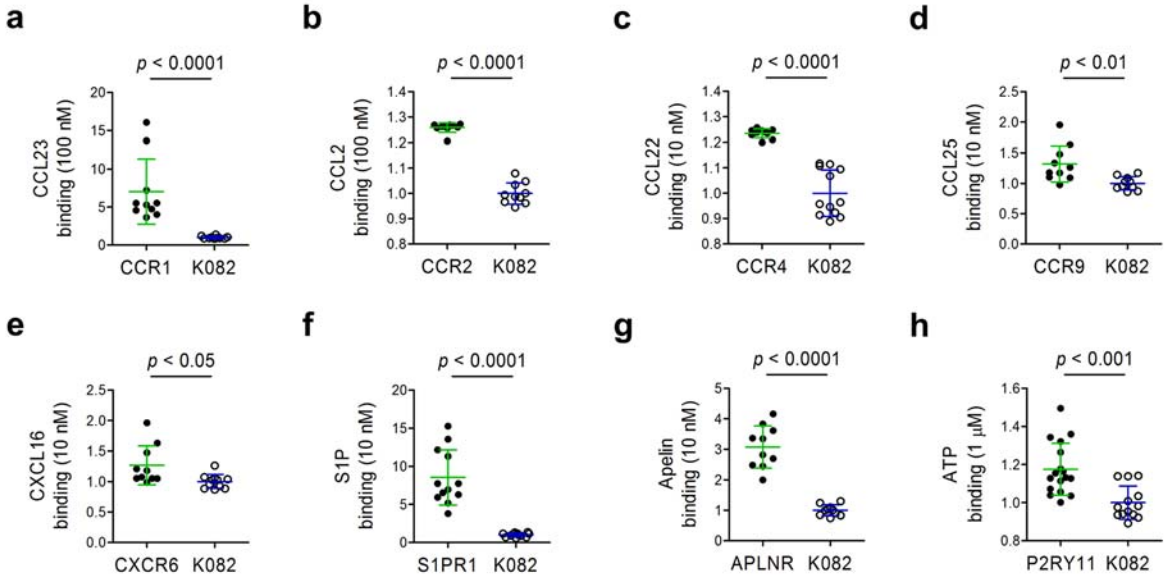

Supplementary Fig. 4 Ligand binding assays with VirD-GPCRs using TIRF microscopy. **a-h** Eight VirD-GPCRs that failed to be recognized by the commercial mAbs were immobilized separately on cover slips and the binding signals of fluorescent-labeled ligands were recorded using TIRF microscopy. K082 virions were used as a negative control in parallel. The relative binding signals were quantified with either integrated intensity or binding event counts and analyzed by Student's *t*-test. Each GPCR measurement was normalized with its corresponding K082 control, which was set to 1. The number of independent measurements, n = 10/10 (**a, b, d, g**), n = 10/12 (**c**), n = 10/11 (**e**), n = 12 (**f**), and n = 17/13 (**h**).

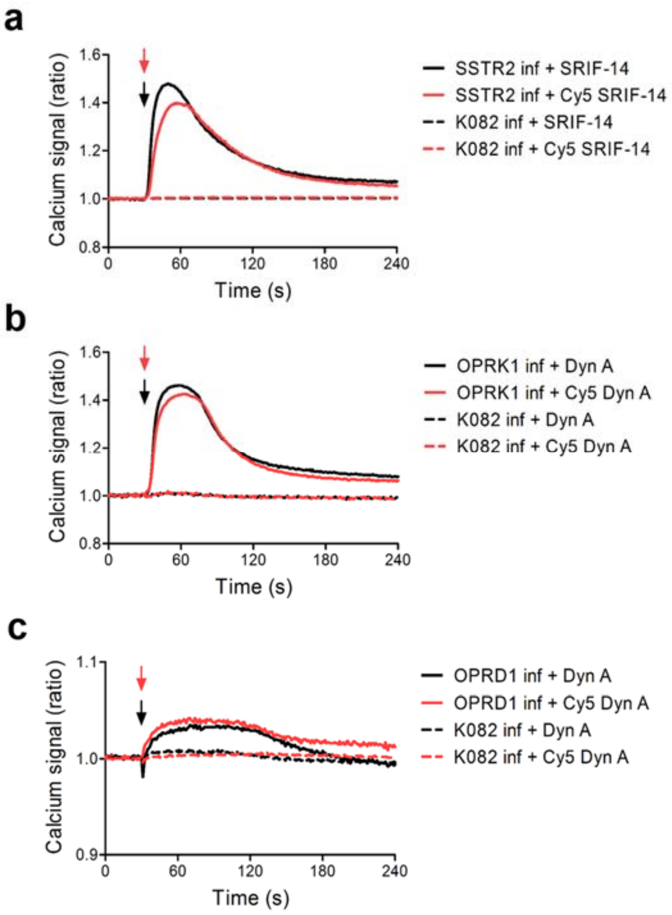

Supplementary Fig. 5 Effects of ligand labeling on GPCR signal transduction. **a-c** Gα-15 cells were infected with GPCR or K082 viruses for 16 h and stained with FLIPR6 calcium indicator (Molecular Devices). The calcium influxes were then monitored at 485 nm / 525 nm before and after adding the fluorescent-labeled or -unlabeled ligands.

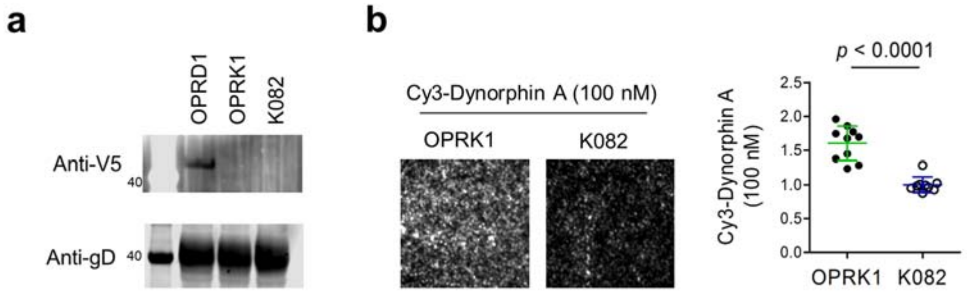

Supplementary Fig. 6 Expression and ligand binding of OPRK1. **a** Comparison of the expression level of OPRK1 and OPRD1 using V5 blot. The anti-gD served as loading control. **b** OPRK1 and K082 were immobilized on cover slips and the binding signals of Cy3-labled Dynorphin A were recorded using TIRF microscopy. The relative binding signals were quantified with either integrated intensity or binding event counts and analyzed by Student's *t*-test. Each GPCR measurement was normalized with its corresponding K082 control, which was set to 1. The number of independent measurements, n = 10/9.
