# Supplementary material for: Development and application of a high-content virion display human GPCR array
Source: Nat Commun. 2019 Apr 30;10:1997. doi: 10.1038/s41467-019-09938-9 (PMC6491619; doi:10.1038/s41467-019-09938-9)
Supplement: Supplementary file 3 — Reporting Summary [file 41467_2019_9938_MOESM3_ESM.pdf]

nature research

Corresponding author(s): Heng Zhu

Last updated by author(s): Mar 28, 2019

Reporting Summary

Nature Research wishes to improve the reproducibility of the work that we publish. This form provides structure for consistency and transparency in reporting. For further information on Nature Research policies, see Authors & Referees and the Editorial Policy Checklist.

Statistics

For all statistical analyses, confirm that the following items are present in the figure legend, table legend, main text, or Methods section.

n/a	Confirmed	
☐	☒	The exact sample size (*n*) for each experimental group/condition, given as a discrete number and unit of measurement
☐	☒	A statement on whether measurements were taken from distinct samples or whether the same sample was measured repeatedly
☐	☒	The statistical test(s) used AND whether they are one- or two-sided<br>*Only common tests should be described solely by name; describe more complex techniques in the Methods section.*
☒	☐	A description of all covariates tested
☐	☒	A description of any assumptions or corrections, such as tests of normality and adjustment for multiple comparisons
☐	☒	A full description of the statistical parameters including central tendency (e.g. means) or other basic estimates (e.g. regression coefficient) AND variation (e.g. standard deviation) or associated estimates of uncertainty (e.g. confidence intervals)
☐	☒	For null hypothesis testing, the test statistic (e.g. *F*, *t*, *r*) with confidence intervals, effect sizes, degrees of freedom and *P* value noted<br>*Give P values as exact values whenever suitable.*
☒	☐	For Bayesian analysis, information on the choice of priors and Markov chain Monte Carlo settings
☒	☐	For hierarchical and complex designs, identification of the appropriate level for tests and full reporting of outcomes
☒	☐	Estimates of effect sizes (e.g. Cohen's *d*, Pearson's *r*), indicating how they were calculated

Our web collection on statistics for biologists contains articles on many of the points above.

Software and code

Policy information about availability of computer code

Data collection	GenePix software, Glomax fluorescent reader, Keyence fluorescence microscopy
Data analysis	Two-tailed Student's t-test were performed by either using Microsoft Excel or Prism GraphPad Software.

For manuscripts utilizing custom algorithms or software that are central to the research but not yet described in published literature, software must be made available to editors/reviewers. We strongly encourage code deposition in a community repository (e.g. GitHub). See the Nature Research guidelines for submitting code & software for further information.

Data

Policy information about availability of data

All manuscripts must include a data availability statement. This statement should provide the following information, where applicable:

- Accession codes, unique identifiers, or web links for publicly available datasets
- A list of figures that have associated raw data
- A description of any restrictions on data availability

The list of GPCR we cloned was attached in the Supplementary Table 2. The datasets generated during and/or analyzed during the current study are available from the corresponding author on reasonable request.

Field-specific reporting

Please select the one below that is the best fit for your research. If you are not sure, read the appropriate sections before making your selection.

☒ Life sciences ☐ Behavioural & social sciences ☐ Ecological, evolutionary & environmental sciences

For a reference copy of the document with all sections, see nature.com/documents/nr-reporting-summary-flat.pdf

Life sciences study design

All studies must disclose on these points even when the disclosure is negative.

Sample size	For studies using TIRF microscopy to measure the ligand binding to the GPCR virions, at least 10 biologically independent samples per group were used. For the ligand binding to the cells, 3 independent samples per group were used. All the sample sizes were described in the associated figure legends.
Data exclusions	No data were excluded from the analyses.
Replication	All attempts at replication were successful. Methods and materials used in our experiments were described in the manuscripts to allow reliable replication of our studies.
Randomization	No randomization was used produce GPCR-VirD array because we need the genetic information. We randomize to pick several mAbs to test on the VirD-GPCR array. We randomize to validate the SRIF-14 ligand bindings. All the results from the array screening were validated with other methods and performed by different people.
Blinding	Blinding was not relevant because we performed multiplex and unbiased screening. The screening results were validated with different methods and performed by different person.

Reporting for specific materials, systems and methods

We require information from authors about some types of materials, experimental systems and methods used in many studies. Here, indicate whether each material, system or method listed is relevant to your study. If you are not sure if a list item applies to your research, read the appropriate section before selecting a response.

Materials & experimental systems

n/a	Involved in the study	
☐	☒	Antibodies
☐	☒	Eukaryotic cell lines
☒	☐	Palaeontology
☐	☒	Animals and other organisms
☒	☐	Human research participants
☒	☐	Clinical data

Methods

n/a	Involved in the study	
☒	☐	ChIP-seq
☒	☐	Flow cytometry
☒	☐	MRI-based neuroimaging

Antibodies

Antibodies used	Monoclonal antibodies reactive to ectodomain of human GPCRs were purchased from R&D system except for P2RY13 which was from ProMab. Anti-HSV-1 gD antibody DL6 was purchased from Santa Cruz Biotechnology.
Validation	Antibodies were validated with IFA and Immunoblot. The displayed targets were validated with ligands.

Eukaryotic cell lines

Policy information about cell lines

Cell line source(s)	Vero, Human Embryonic Lung, HeLa, and HEK-293T cells were available in ATCC.
Authentication	Purchased from ATCC.
Mycoplasma contamination	Free of mycoplasma contamination.
Commonly misidentified lines (See ICLAC register)	None.

Animals and other organisms

Policy information about studies involving animals; ARRIVE guidelines recommended for reporting animal research

Laboratory animals	BALB/C strain.
Wild animals	None.
Field-collected samples	None.
Ethics oversight	All procedures and handling techniques were approved by the Animal Care and Use Committee of the Johns Hopkins University.

Note that full information on the approval of the study protocol must also be provided in the manuscript.
